# Structural and functional MRI evidence for significant contribution of precentral gyrus to flexible oculomotor control: evidence from the antisaccade task

**DOI:** 10.1007/s00429-022-02557-z

**Published:** 2022-09-01

**Authors:** Zhenlan Jin, Dong-gang Jin, Min Xiao, Aolin Ding, Jing Tian, Junjun Zhang, Ling Li

**Affiliations:** MOE Key Laboratory for Neuroinformation, High-Field Magnetic Resonance Brain Imaging Key Laboratory of Sichuan Province, Center for Psychiatry and Psychology, School of Life Science and Technology, University of Electronic Science and Technology of China, Chengdu, 610054 China

**Keywords:** Antisaccade cost, Gray matter volume, Precentral gyrus, Insula, Structural MRI, Task-state functional MRI

## Abstract

Antisaccade task requires inhibition of a prepotent prosaccade to a peripheral target and initiation of a saccade to the opposite location, and, therefore, is used as a tool to investigate behavioral adjustment. The frontal and parietal cortices are both known for their activation during saccade generation, but it is unclear whether their neuroanatomical characteristics also contribute to antisaccades. Here, we took antisaccade cost (antisaccade latency minus prosaccade latency) as an index for additional time for generating antisaccades. Fifty-eight participants conducted pro and antisaccade tasks outside the magnetic resonance imaging (MRI) scanner and their structural MRI (sMRI) data were also collected to explore brain regions neuroanatomically related to antisaccade cost. Then, twelve participants performed saccade tasks in the scanner and their task-state functional MRI (fMRI) data were collected to verify the activation of structurally identified brain regions during the saccade generation. Voxel-based morphometry (VBM) results revealed that gray matter volume (GMV) of the left precentral gyrus and the left insula were positively correlated with the antisaccade cost, which was validated by the prediction analysis. Brain activation results showed the activation of the precentral during both pro and antisaccade execution period, but not the insula. Our results suggest that precentral gyrus and insula play vital roles to antisaccade cost, but possibly in different ways. The insula, a key node of the salience network, possibly regulates the saliency processing of the target, while the precentral gyrus possibly mediates the generation of saccades. Our study especially highlights an outstanding role of the precentral gyrus in flexible oculomotor control.

**Supplementary Information:**

The online version contains supplementary material available at 10.1007/s00429-022-02557-z.

## Background

In everyday life, human beings dynamically adjust their behaviors for a better adaption to constantly changing environments, enabled by cognitive control (Bissett and Logan 2011). Eye movements are frequently used to explore visual brain function including flexible control over behaviors. Antisaccade task is a classical and important task to study flexible oculomotor control. The antisaccade task was first introduced by Hallett as a “novel task” (Hallett 1978) where participants were required to inhibit a prepotent prosaccade to a peripheral target and generate a saccade to the mirror location of the target instead. Thus, it is frequently used to investigate the ability to suppress reflexive responses in favor of voluntary motor actions. Compared with prosaccades, antisaccades have longer latency and higher error rate (Forbes and Klein 1996; Fischer and Weber 1997; Everling and Fischer 1998). The prolonged latency is often referred to as the antisaccade cost (Liu et al. 2010; Jóhannesson et al. 2013). It has been argued that suppression of the reflexive prosaccade and generation of a saccade in the opposite direction are important for the antisaccade task (Everling and Fischer 1998), which may explain the prolonged latency of antisaccades. The suppression of the reflexive prosaccade is related to the stage of saccade programming, supported by studies using a double-step saccade task which adapted a go-nogo paradigm (Ray et al. 2004; Emeric et al. 2007; Nelson et al. 2010). In this type of double-step saccade task, sequentially presented two targets require participants to cancel a preprogrammed initial saccade to the first target and directly make a saccade to the second target instead. Temporal delay between the two sequential targets determines the difficulty of inhibiting a saccade toward the initial target as studies found bigger difficulty for longer temporal delay, which indicates that the suppression of the initial saccade becomes easier and requires less cognitive control if the reflexive initial saccade generation posits at an earlier stage of programming (Ray et al. 2004; Emeric et al. 2007; Nelson et al. 2010). Since saccade generation is closely bound with attention shift and the attention shifts to the target 50–150 ms before the saccade initiation (Kowler et al. 1995; Deubel 2008), attention shift to the target is critical to the programming stage of the saccade. Hence, attention also plays a role in the generation of antisaccades.

Human imaging studies have indicated that saccadic eye movements recruit brain regions including the frontal eye field (FEF), supplementary eye field (SEF), posterior parietal cortex (PPC), insula, superior colliculus and so on (Sweeney et al. 1996; Müri et al. 1998; Tobler et al. 2001; Krebs et al. 2010; Hu and Walker 2011). Specifically, the FEF, the SEF and the parietal eye field (eye movement related region within the intraparietal sulcus of the PPC) are known as three main cortical eye fields (Pierce and McDowell 2016). Among these brain regions, the FEF stands out for its importance in saccade initiation and higher activity during antisaccades than during prosaccades (Cornelissen et al. 2002; Connolly et al. 2005). Connolly et al. (2005) found that the FEF and SEF, but not the intraparietal sulcus showed preparatory activity before the saccade target onset. Moreover, only the FEF showed its preparatory activity correlated with saccade latencies (Connolly et al. 2005). Anatomically, the FEF posits in the precentral gyrus. Thus, some studies referred to saccade related brain regions using the anatomical name of brain regions as well. For example, Curtis and Connolly (2008) found superior and inferior precentral sulcus, paracentral sulcus, and intraparietal sulcus were activated during both prosaccades and antisaccades. Additionally, the superior precentral sulcus was activated more in antisaccade trials than in prosaccade trials when preparing saccades, and this region also showed higher activity in the antisaccade trials during the saccade execution period (Curtis and Connolly 2008). Similarly, Pierce and McDowell (2016) found that brain regions including bilateral precentral gyrus, medial frontal gyrus (SEF/ACC), bilateral precuneus, etc. showed higher activation during antisaccades than during prosaccades. Together, the precentral area or the FEF seems to be critical for antisaccades although distributed brain regions are involved in saccade generation. However, few studies checked whether neuroanatomical characteristics of the frontal also contributed to antisaccades.

Brain–behavior relationship considers individual differences and it has been successfully found that neuroanatomical features are related to the performance of various cognitive functions (Kanai et al. 2011; Xu et al. 2014; Tadayon et al. 2020; Xie et al. 2020). For example, gray matter volume (GMV) obtained from voxel-based morphometry analysis (VBM) is considered as an indicator of brain structural changes (Ashburner and Friston 2000). Smaller GMV in the medial prefrontal cortex, bilateral precentral and postcentral gyrus, insula, and subcortical areas is related to higher cognitive flexibility, consistent with findings of task-related functional MRI study (Weise et al. 2019). These studies investigated the brain-behavior relationship via considering individual’s difference and confirmed these methods are effective to explore the neural bases underlying individual difference in cognitive functions. Regarding the relationship between antisaccades and gray matter volume, Ettinger et al. (2005) found that the gray matter volume of the right middle frontal gyrus negatively correlated with antisaccade error rate, but no brain regions survived correction for multiple correlations when checking the correlation between the GMV and antisaccade latencies.

The present study aimed to investigate brain regions whose anatomical features are associated with individual differences of initiation of antisaccades and check their involvements during the saccade generation. To achieve this, we collected eye movements data of the prosaccade and antisaccade tasks and T1-weighted structural MRI for each participant. For saccadic task performance, we measured the error rate and the saccadic latency. The antisaccade cost was computed for every participant to reflect the additional time for suppressing the reflexive prosaccade and generating an antisaccade by subtracting the latency of prosaccades from that of antisaccades. We used VBM analysis to explore brain regions whose neuroanatomical features are correlated with individual differences of the antisaccade cost. Considering that the additional processes of antisaccades may lead to longer latency, we expect to find brain regions related to these additional processes such as attention and those related to eye movements. To further validate the role of the structurally identified brain regions in the antisaccade cost, the task-state fMRI data were also collected to check brain activation during the saccade generation. 

## Method

### Participants

Fifty-eight right-handed naïve students (27 male, age mean 22.22 years, SD 2.29) from University of Electronic Science and Technology of China (UESTC) were recruited for the main experiment and twelve of them also participated in the task-related fMRI experiment. All participants had normal or corrected-to-normal vision and none of them had a history of neurological diseases or contraindications to MRI scans (e.g., dental plates). This research was conducted under the Declaration of Helsinki and approved by the Magnetic Resonance Imaging (fMRI) Research Center in the UESTC and the Human Body Protection Board. All participants signed written informed consent and were compensated for the participation.

### Eye movement data acquisition

Eye movements data were collected by the EyeLink 1000 Plus system (SR Research Ltd., Canada) at a sampling rate of 2000 Hz. Visual stimuli of the eye movement experiment were presented using E-Prime 1.0 (Psychology Software Tools, Pittsburgh, PA, USA) a display with 1024 × 768 pixels resolution at the refresh rate of 60 Hz. The participants viewed the stimuli binocularly, but only the right eye positions were recorded. The participants conducted eye movement experiment in a dim, quiet and enclosed room and the distance between the participants eye and stimuli display was 55 cm with the help of a chin and forehead rest.

## Experiment procedure

**Fig. 1:**
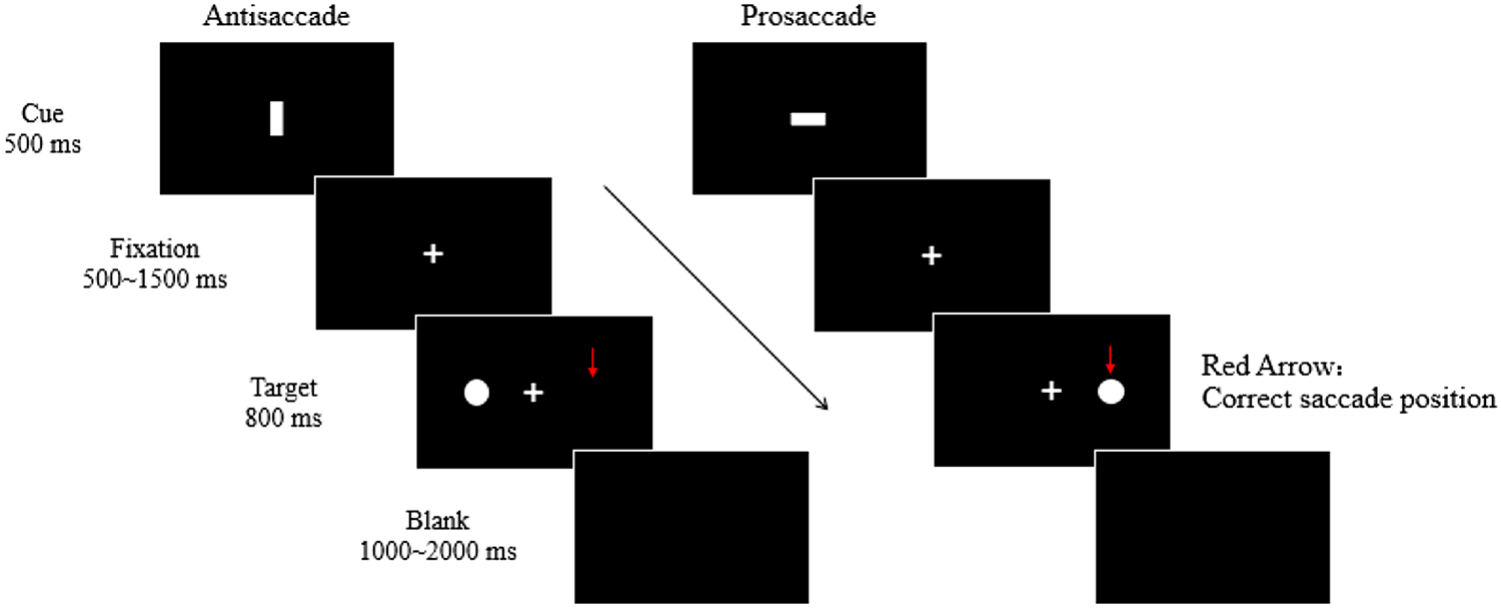
Schematic trial procedure in the main experiment. At the start of a trial, a cue (horizontal or vertical bar) appeared and indicated the type of saccade. Upon the target onset, the participants were required to make a saccade toward the target or toward the mirror location of the target according to the cue (horizontal bar for prosaccade, vertical bar for antisaccade). Here, the red arrow indicates correct saccade landing position for the illustrated trials

In the main experiment, each trial started with a display of a horizontal or vertical white bar [RGB (255, 255, 255)] for 500 ms which indicated the type of saccade, horizontal bar (1.82° × 0.7°) for prosaccade task and vertical bar (0.7° × 1.82°) for antisaccade task (see Fig. 1). After the cue, a 500–1500 ms of white fixation cross [1.39° × 1.39°, RGB (255, 255, 255)] appeared at the display center and it was followed by a peripheral saccadic target, a white disk [1.39° × 1.39°, RGB (255, 255, 255)]. The target appeared at the left or right horizontal meridian randomly with an eccentricity of 11.21° and stayed on the display for 800 ms. Upon the target onset, the participants were required to make a saccade quickly toward the target if the cue was a horizontal bar (prosaccade trial) or make a saccade quickly toward the mirror location of the target if the cue was a vertical bar (antisaccade trial). There was a random intertrial interval display (1000–2000 ms). Prosaccade (24 trials) and antisaccade (24 trials) trials were presented in a block randomly. Every participant practiced one block to get familiar with the task requirement. For the main experiment, each participant conducted four blocks in total with 96 prosaccade trials and 96 antisaccade trials. Between blocks, the participants took several-minutes break to avoid fatigue.

In the task-related fMRI experiment, the subjects performed saccade tasks in the scanner and their task-related fMRI data were collected while they conducted the saccade tasks. In this experiment, the same visual stimuli were used and the trial procedure was basically the same as the main experiment except that the duration of the fixation, target, and blank display were extended to 1500/2500/3500 ms, 1000 ms, 2000/3000 ms respectively. Thirty-two trials (16 prosaccade and 16 antisaccade trials) comprised a block and every block took about three and half minutes. Each subject conducted three blocks and took a five minutes break between blocks to avoid fatigue.

### MRI acquisition

The MRI data were acquired using a GE Sigma 3.0 T scanner (General electric, Milwaukee, WI, USA) equipped with an eight-channel head coil at the MRI Brain Imaging Center. In this study, high-resolution T1-weighted structural MRI and task-state functional MRI (fMRI) data were acquired with the following parameters. The parameters of T1-weighted images were TR = 5.96 ms, TE = 1.96 ms, FA = 9°, FOV = 256 × 256 mm^2^, voxel size = 1 × 1 × 1 mm^3^, and 176 slices with slice thickness of 1.0 mm and no gap. Echo planar task-state fMRI images were acquired using the following parameters: TR = 2000 ms, TE = 30 ms, FA = 90°, FOV = 240 × 240 mm^2^, matrix size = 64 × 64, voxel size = 3.75 × 3.75 × 3 mm^3^, slice thickness/gap = 3.75 mm/0.6 mm and 43 slices in total. During the task-state fMRI scan, the participants conducted saccade tasks and data of 318 repetition times were collected in total. During the T1 scan, the participants stayed awake and lay still with their eyes closed.

### Structural MRI data analysis

Preprocessing of the high-resolution T1-weighted images for VBM was performed using the Statistical parametric mapping toolbox 12(SPM 12; https://www.fil.ion.ucl.ac.uk/spm/) and the Computational Anatomy Toolbox 12(CAT 12; http://dbm.neuro.uni-jena.de/cat12/; (Gaser and Dahnke 2016). The preprocessing steps were based on the CAT12 manual, including:(1) visual check for artifacts and orientation of raw images, (2) segmentation into gray matter, white matter, and cerebrospinal fluid, (3) spatial normalization to the Montreal Neurological Institute space using high-dimensional DARTEL template and resample to 1.5 × 1.5 × 1.5 mm^3^, (4) estimating the total intracranial volume(TIV) to subsequently correct for different brain sizes and volumes across participants, (5) spatial smoothing using an 8 mm full-width-half-maximum Gaussian kernel.

Multiple regression analysis was conducted to explore brain regions whose GMV correlated with individual antisaccade cost, respectively. Age, gender and TIV were controlled for as nuisance parameters. Before removing the variance explained by TIV, we inspected the coefficient of association between antisaccade cost and TIV (*r* = −0.1955, *p* = 0.1606). The GMV analysis were masked with an absolute threshold of 0.1 to eliminate gray-white matter boundary effects, in which voxels with gray matter or white matter values under 0.1 were excluded from the analysis. For the voxel-wise analysis, results were corrected for multiple comparisons using a peak threshold of *p* < 0.05 (FDR corrected) and a cluster size of > 30 voxels.

Among the clusters obtained from the structural MRI analysis, we selected brain regions using cluster-wise FWE-correction as a criteria performed leave-one-subject-out cross-validation analyses and checked the predictability of the GMV of all brain regions to the antisaccade cost. Furthermore, 5000 iterations permutation test (two-tailed) was used to validate the brain-behavior relationship.

### Task-state fMRI data analysis

Preprocessing of the task-state fMRI data was performed using the Data Processing Assistant for Resting-State fMRI Advanced Edition (DPARSFA, http://rfmri.org/DPARSF; (Yan and Zang 2010). For each subject, the first 5 volumes were discarded for magnetization equilibrium. Subsequent preprocessing included slice timing, head motion correction, registration to the anatomical image of the individual, spatial normalization to the MNI template, resampling to 3 × 3 × 3 mm^3^, and smoothing using an 8 mm Gaussian kernel.

Statistical analysis of fMRI data was performed using statistical parametric mapping software (SPM12, http://www.fil.ion.ucl.ac.uk/spm). To obtain task-state activation results for different response phases in different trial types, for the individual level analysis, we used both trial types (prosaccade, antisaccade) and two phases (cue, saccade target) as regressors when building the general linear model, and six head movement parameters were treated as regressors of no interest. With the blank phase serving as the activity baseline, we modeled the onset the cue to the end of the fixation as the cue phase. The activation of four conditions (cue and saccade target phases in both prosaccade and antisaccade trials) at the individual level was obtained from the first-level analysis. The results of the first-level analysis were then analyzed at the second level and significant activation regions at the group level were obtained by a one-sample *t *test. Significance levels were set at *p* < 0.05 (FWE corrected) and a minimum cluster size of 10 voxels.

### Eye movements data analyses and statistical analysis

Eye movements’ data were analyzed offline and saccade detection used the algorithm provided by Eyelink 1000 system, with the velocity threshold of 30°/s and acceleration threshold of 4000°/s^2^. Only the first saccade after the target onset was regarded as the saccadic response for the trial. Trials meeting any of the following criteria were excluded for the analysis in both experiments: (1) trials with blink since 50 ms before the target onset to saccade initiation; (2) trials with a saccade occurring within 50 ms time window before the target onset; (3) eye positions deviated from the fixation cross more than 3° in the horizontal meridian; (4) the amplitude of the first saccade of less than 3°; (5) latency beyond three standard deviations for each condition. With these criteria, one subject with less than ten trials was excluded. Another four subjects were excluded because their latency or error rate of saccades were beyond three standard deviations, which resulted in the inclusion of 53 subjects’ data for further analysis. In the main experiment, 91.8% of prosaccade trials and 91.5% of antisaccade trials were retained for further analysis. In the task-state fMRI experiment, 86.6% of prosaccade trials and 87.7% of antisaccade trials were retained for further analysis.

For the statistical analysis of eye movements data, paired *t* test was conducted to compare the latency and error rate between the prosaccades and antisaccades. Then, the antisaccade cost was computed by subtracting the prosaccade latency from the antisaccade latency and the difference of error rates was computed by subtracting error rate of the prosaccades from that of the antisaccades. Kolmogorov–Smirnov test was followed for normality of the antisaccade cost and difference of error rates between prosaccades and antisaccades.

## Results

### Eye movements results

We found that the antisaccade trials showed longer latency [*t*(52) = −13.572, *p* = 0.000] and higher error rate [*t*(52) = 7.068., *p* = 0.000] than prosaccade trials (see details in Table 1), consistent with common findings about antisaccades. Average antisaccade cost (antisaccade latency minus prosaccade latency) was 56.34 ± 30.22 (mean ± SD) ms and average error rate difference (prosaccade error rate minus antisaccade error rate) was 8.88 ± 9.14% (mean ± SD). Both followed normal distribution (Kolmogorov–Smirnov test, *p* = 0.200), enabling reliable VBM analysis.

**Table 1:** Latency and error rate of saccades

	Prosaccade (mean ± SD)	Antisaccade (mean ± SD)
Latency (ms)	310.47 ± 41.37	366. 81 ± 39.31
Error rate (%)	10.89 ± 5.69	19.77 ± 10.09

### Results of VBM analyses and predictive analysis

**Fig. 2:**
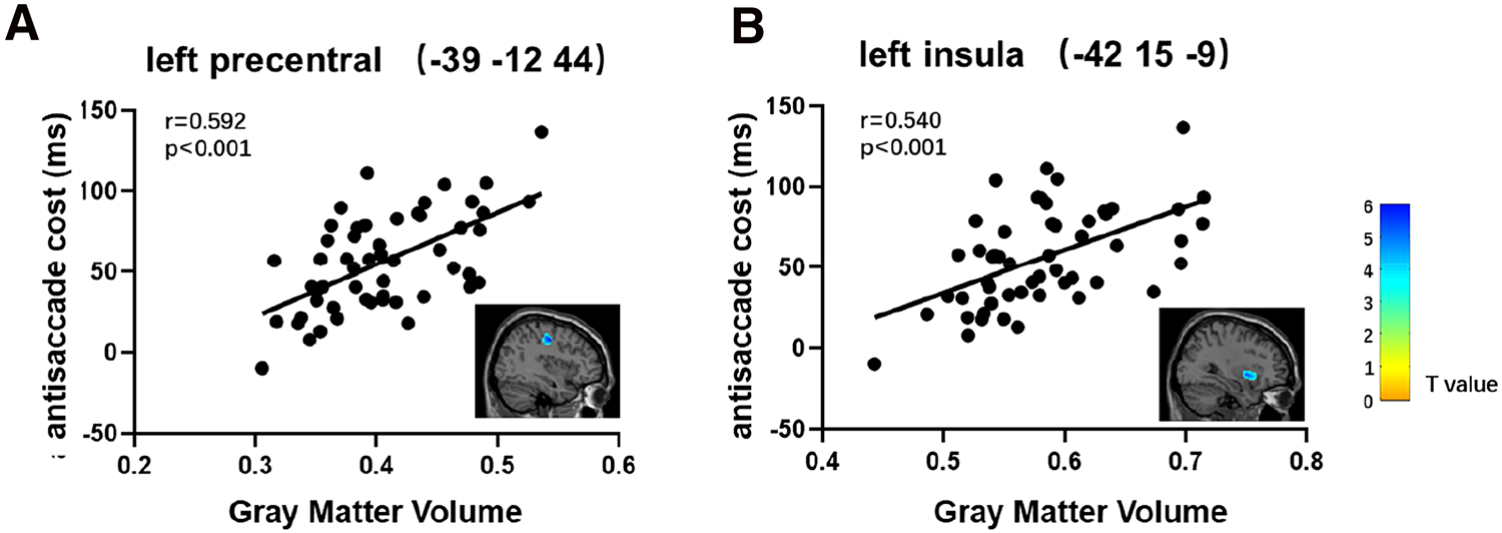
Results of the VBM analysis. The GMV of the left precentral (**A**) and the left insula (**B**) were positively correlated with the antisaccade cost after regressing out age, gender, and TIV

**Fig. 3:**
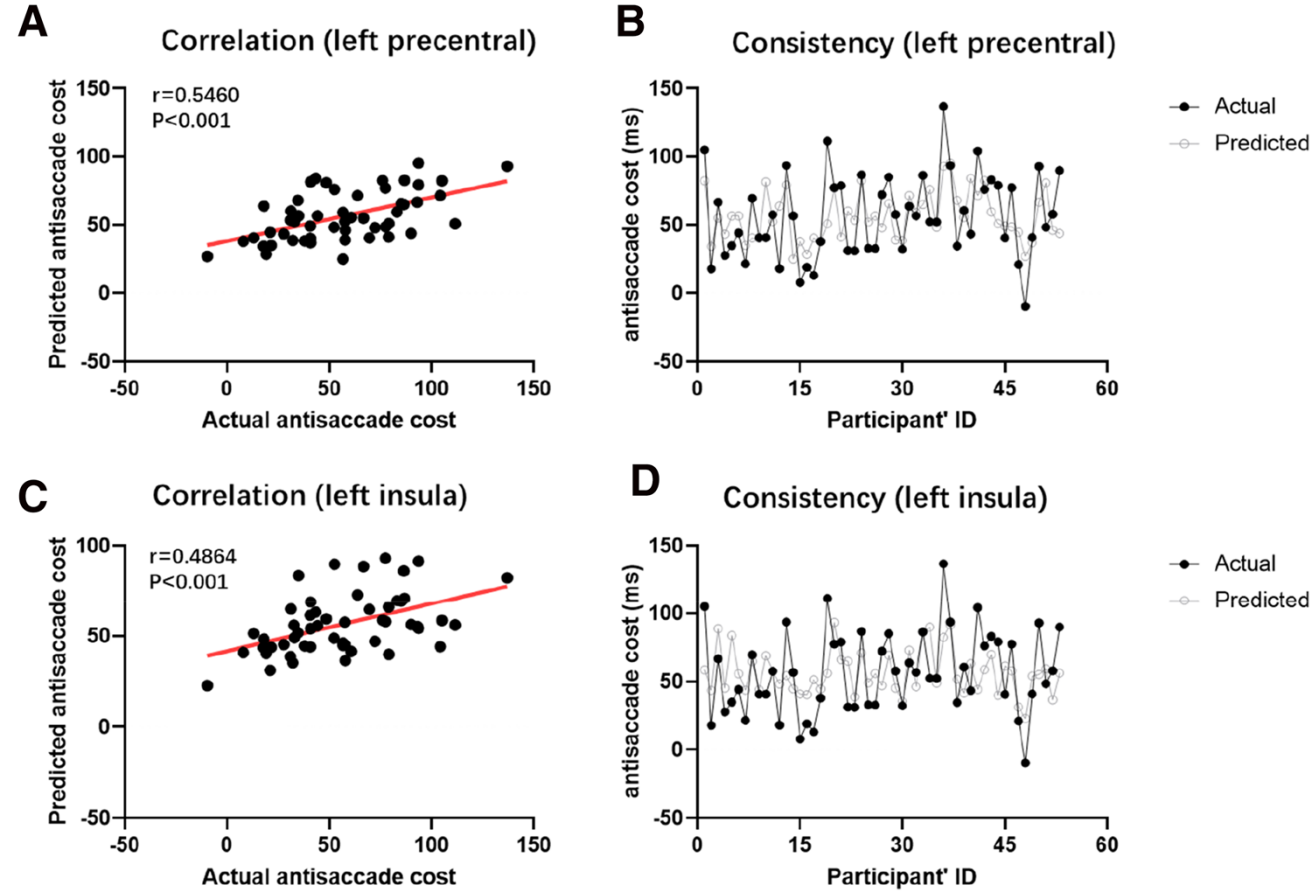
Results of the predictive analysis. Individual's antisaccade cost was successfully predicted by the GMV of the left precentral (**A**, **B**) and the left insula (**C**, **D**)

The VBM analysis showed that the antisaccade cost was positively correlated with the GMV in the left precentral gyrus [peak MNI = (−39 −12 44), *r* = 0.592, Fig. 2A] and left insula [peak MNI = (−42, 15, −9), *r* = 0.540, Fig. 2B]. Detailed information is shown in Table 2. The clusters were named after the brain region where the most of the voxels in the cluster belonged. In the left precentral cluster, the precentral gyrus occupied 83.8% (528/630) of the cluster. In the left insula cluster, the insula occupied 56.9% (518/911) of the cluster. Leave-one-out cross-validation analysis (Fig. 3) revealed that the GMV of the left precentral and the left insula could predict the antisaccade cost (precentral: *r* = 0.5460, insula: *r* = 0.4864), and after breaking the true brain-behavior relationship, both prediction results passed permutation test (iterations = 5000, *p* < 0.001). These results demonstrated that the gray matter volume of both the left postcentral and left insula contribute to individual differences in the latency cost of antisaccades (Fig. 3).

**Table 2:** Brain regions whose GMV was positively correlated with antisaccade cost

Brain region	Cluster (voxels)	Peak MNI coordinates			*T *value	*r*_cluster_^a^
		*x*	*y*	*z*		
L precentral	630	−39	−12	44	5.77	0.592^b^
		−50	−12	53		
L insula	911	−42	15	−9	5.19	0.540^b^
		−32	6	−3		

*MNI* Montreal Neurological Institute; *L* left, *R* right

^a^The correlation coefficient between the averaged GMV within the significant clusters and the antisaccade cost

Significance levels were set at of *p* < 0.05 (FDR corrected) and a minimum cluster size of 30 voxels. ^b^*p* < 0.001

GMV in no brain region was significantly correlated with error rates of prosaccades and antisaccades, and the difference of error rates between two saccade type trials.

**Fig. 4:**
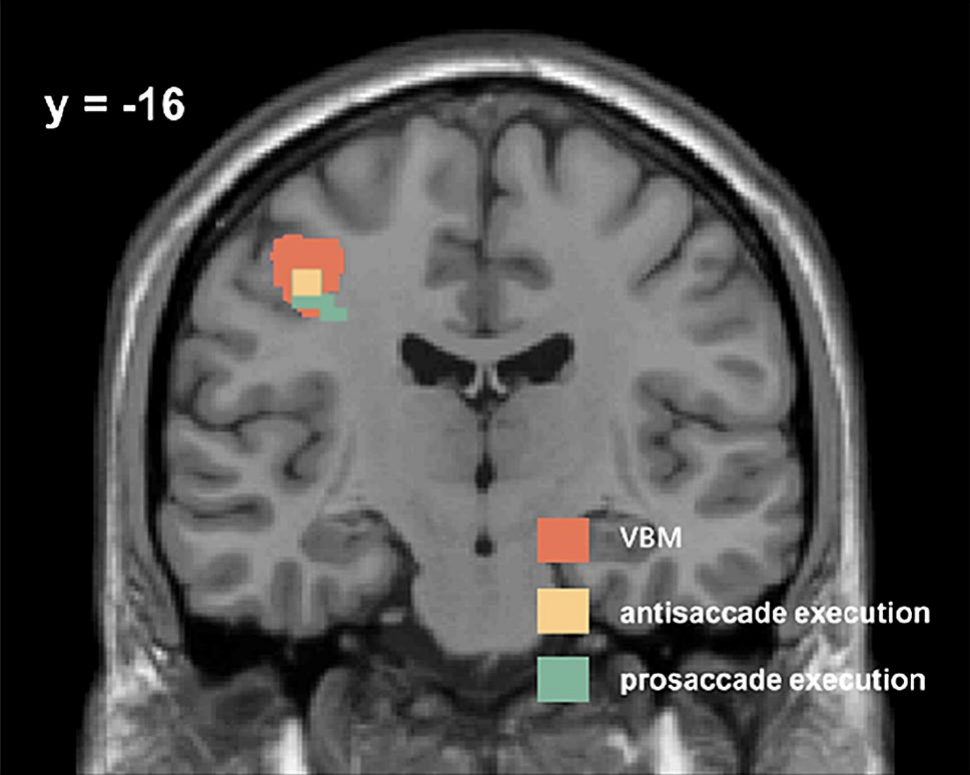
The left precentral areas found in the VBM analysis were also activated during the saccade execution phase. The yellow [peak MNI: (−42 −15 45)] and green [peak MNI: (−42 −15 42)] brain regions show the activated areas in the saccade execution phase were close to the structurally identified precentral gyrus

The eye movements data collected in the scanner showed longer latency [*t*(11) = −5.982, *p* = 0.000] and higher error rate [*t*(11) = 6.643, *p* = 0.000] in the antisaccade task than in the prosaccade task. Task-state fMRI data analysis did not show any significant brain activation during the cue period, possibly due to the small sample size. However, we found that several brain regions in the frontal and parietal lobes were activated in the saccade target period, demonstrating that these brain regions were involved in the saccade execution period (see details in Table 3). We can see from Table 3 that brain regions around the left precentral obtained from the VBM analysis [MNI: (−39 −12 44)] were activated during the execution phase of the prosaccades and antisaccades (prosaccade, MNI [−42 −15 42] and antisaccade, MNI [−42 −15 45]). Figure 4 showed that the brain regions activated during the saccade execution phase were largely overlapped with the left precentral whose GMV was correlated with the antisaccade cost, suggesting that neuroanatomically identified left precentral was important in both prosaccade and antisaccade execution. No brain areas showed significant difference of activation between prosaccades antisaccades during the execution period.

**Table 3:** Brain regions that were activated during the execution of prosaccades and antisaccades

Brain region	Cluster (voxels)	Peak MNI coordinates			*T* value
		*x*	*y*	*z*	
Prosaccade					
Left frontal	38	−33	−12	36	13.00
		−42	−15	42	
Right precentral	16	36	−6	48	10.66
		36	−12	48	
Antisaccade					
Left inferior parietal	12	−33	−48	54	11.93
Left postcentral	11	−42	−15	45	11.15

*MNI *Montreal Neurological Institute, *L* left, *R* right

Significance levels were set at of *p* < 0.05 (FWE corrected) and a minimum cluster size of ten voxels

In addition, we checked the relationship between the structural feature and functional activity of the structurally identified precentral and insula by conducting correlation analysis. To do it, we extracted the beta value of the precentral and insula during cue and target periods in both prosaccade and antisaccade conditions. Pearson correlation results showed that the GMV in the insula was positively correlated with the beta value of the target period in prosaccade condition [*r* = 0.628, *p* = 0.028; confidence interval: (0.152 0.920) based on bootstrap 5000 iterations], indicating the link between the GMV and functional activity. These results suggest that individuals with bigger GMV show higher activation during the prosaccade execution phase and it may serve as evidence for the involvement of the insula in saccade generation to some extent.

## Discussion

The present study investigated neuroanatomical bases of the antisaccade cost, especially the neuroanatomical contribution of the frontal and parietal areas which are widely known for their functional roles in eye movements. We first explored brain regions neuroanatomically associated with the antisaccade cost based on structural MRI and brain regions functionally contributing to the saccade generation based on task-state fMRI. We found that (1) the GMV of the left precentral gyrus and the left insula were positively correlated with the antisaccade cost and both could successfully predict an individual’s antisaccade cost; (2) only the left precentral was significantly activated during the prosaccade and antisaccade execution period. Based on these results, we propose that the left precentral contributes to individual difference of the antisaccade cost via modulating the execution of saccades. Considering the abrupt onset of the saccade target in the current design, the insula, a key node of salience network, may be associated with the saccade target processing via modulating the saliency of the saccade target.

Antisaccade task showed prolonged latency and higher error rate as compared with prosaccade task, replicating common findings (Munoz and Everling 2004; Talanow et al. 2020). Compared with prosaccade task, antisaccade task requires additional effort to inhibit prepotent saccade to the target and instead generates a saccade to the mirror location of the target. Hence, smaller antisaccade cost indicates better ability of flexible oculomotor control. These additional processes may be responsible for the prolonged latency of antisaccades (Everling and Fischer 1998). In the current experimental design, the saccade target was abruptly presented and the participant decided the direction and location of the antisaccade according to this target. Saccade generation is closely bound with attention shift and attention shift occurs before the initiation of the saccade (Kowler et al. 1995; Deubel 2008). Since abrupt onset of visual stimulus captures attention (Sunny and von Mühlenen 2013; Theeuwes 1991; Yantis and Jonides 1984) the onset of saccade target in the current study captures attention and may initiate the process of attention shift to the target. The difficulty of inhibiting a saccade is related to its programming stage that a saccade in its early programming stage is easier to inhibit compared with one in latter stage (Ray et al. 2004; Emeric et al. 2007; Nelson et al. 2010). Therefore, in the current study, individual difference of attention capture by the saccade target may further influence the difficulty of inhibiting the reflexive prosaccade in the antisaccade trials. Consistently, it has been reported that attention reorienting is critical for the antisaccade cost (Liu et al. 2010). Therefore, brain areas involved in the additional processing for antisaccades may contribute to the antisaccade cost.

Voxel-based morphometry (VBM) analysis revealed that gray matter volume (GMV) of the left precentral gyrus was positively correlated with the antisaccade cost, which was verified by further leave-one-out cross-validation analysis. The GMV of the left precentral could successfully predict an individual’s antisaccade cost. Individuals less precentral GMV showed better flexible control of the oculomotor system. No parietal regions were found to be related to the antisaccade cost, emphasizing the importance of the frontal cortex in the antisaccades. Structurally found precentral in the present study (peak MNI [−39 −12 44], cluster size 630) was close to the superior precentral sulcus and the left FEF in the previous studies (Connolly et al. 2005; Curtis and Connolly 2008). In the study by Connolly et al. (2005), they identified the left and right frontal eye field (FEF) using a blocked-design localizer task, and right and left FEF were [24 −9 45] and [–27 −14 45] in Talairach coordinates. Previous task-state fMRI study found that the superior precentral sulcus [known cue: MNI (−30.1, −13.3, 49.9); unknown cue: MNI (−31.0, −14.1, 51.5)] showed higher activation during antisaccades than prosaccades in the cue periods (Curtis and Connolly 2008). Combined with previous studies, it could be drawn that the left precentral in the present study seems to be related to the generation of saccades, which further shows functional difference between antisaccades and prosaccades. Importantly, the task-state fMRI results showed nearby cluster were significantly activated during the execution period of both prosaccades and antisaccade. Thus, the left precentral gyrus identified by the VBM analysis may be critical in saccade generation and contributes to the antisaccade cost via influencing saccade execution. In other words, the left precentral is functionally important in saccade execution and its neuroanatomical characteristics is critical in determining individual’s difference of antisaccade cost.

Besides the left precentral gyrus, the GMV of the left insula was also identified by the VBM analysis, indicating its role in the antisaccade cost. The insula is a key node of the salience network which extracts the most relevant information from internal and extrapersonal stimuli to guide behaviors and plays a critical role in high-level cognitive control and attentional processes (Sridharan et al. 2008; Menon and Uddin 2010; Uddin 2015). Especially, the anterior part of the insula is involved in stimulus-driven, bottom-up control of attention (Corbetta et al. 2002, 2008). The insula identified by the VBM analysis was peaked at [−42 15 −9] in MNI coordinate system, which is located at the anterior part. Additionally, the abrupt onset of the saccade target in the current study could trigger the detection of salient events in a bottom-up manner and lead to following processes of saccade generation, including attention shift to the target and saccade initiation. Therefore, individual differences of the GMV in the insula may be related to discrepancies in the target processing across individuals and further affect the inhibition of the prepotent prosaccade to the target via regulating the attention shift which occurs before the saccade initiation. That is, an individual with bigger GMV of the insula may have better or faster attention capture by the target, and thus faster attentional shift to the target location. This may accelerate the reflexive prosaccade generation, resulting in higher difficulty in suppressing reflexive prosaccades in the antisaccade trials. Moreover, the task-state fMRI results showed that the left insula was not significantly activated during the saccade execution phase, but the GMV in the insula was positively correlated with its activation in the prosaccades, indicating the link between the GMV and function. We think the insula mediates the saccade generation via regulating attention shift However, the role of the insula in the antisaccade cost may be limited to the similar experimental design where the saccade target can capture attention in a bottom-up manner. Still, due to a rather small sample size of the task-state fMRI experiment, the activation we observed may be limited, so future study with a bigger sample size may be considered to find related brain regions during the saccade preparation phase.

The present study showed that the GMV in the precentral and insula was associated with the antisaccade cost, but no brain region whose GMV was associated with the antisaccade error rate was found. Using the VBM analysis, a previous study (Ettinger et al. 2005) found the GMV in the right middle frontal gyrus was associated with antisaccade error rate, but did not find latency associated brain regions. Briefly, these results seem inconsistent with the current findings. We think there may be two possible reasons. Firstly, bigger sample size in the present study helped us reveal the antisaccade cost associated brain regions, while Ettinger et al. (2005) did not find any latency associated brain regions. At the same time, if the sample size was the only reason for inconsistency between studies, we would expect to find the brain regions associated with the error rate as well. Yet, no brain regions were found to be associated with the error rates. This may be related to the significance level used in two studies differed. The present study considered brain regions only surviving for the whole brain correction, while the previous study (Ettinger et al. 2005) applied small-volume corrections in the interested brain regions. Differential method of correction possibly leads to different results and we believe the whole brain correction may provide even more robust and reliable findings. When we tried a less strict criteria (uncorrected *p* < 0.001), we found some brain regions associated with the error rates of the prosaccades and antisaccades including the right frontal middle gyrus which was found to be associated with the antisaccade error rate as in the study of Ettinger et al. (2005) (see Table S1 in supplementary materials). Thus, we argue that the present study and Ettinger et al.’s study (2005) can compensate for each other and together suggest that distinct brain regions contribute to antisaccade error rate and latencies within the saccade generation network.

Main findings of the present study were based on the VBM analysis which is a kind of association analysis, so it is inconclusive about the causal contribution of the structurally identified brain regions. Additionally, the present study cannot explain why individuals with higher GMV in the precentral and insula produce bigger antisaccade cost. Pruning or myelination may cause smaller GMV. It has been proposed that less gray matter volume in the cortex is a consequence of synaptic pruning (Kanai and Rees 2011; Gogtay et al. 2004). The number of neurons reduces during the brain maturation and the computational efficiency of cortex increases after the pruning of the cortex (Gogtay et al. 2004; Chechik et al. 1999). The pruning of the cortex may lead to decrease of gray matter volume, thus smaller gray matter volume is possibly related to increased functional specificity of cortical columns (Tadayon et al. 2020). High myelination may lead to incorrect segmentation of gray matter as white matter, resulting in cortical thinning (Natu et al. 2019). Since gray matter volume can be decomposed of cortical thickness and surface area (Winkler et al. 2010), high myelination may cause smaller gray matter volume. In addition, the relationship between gray matter and function is still unclear, though a variety of studies verified the roles of brain regions in cognitive functions by jointly using structural and functional MRI (Lin et al. 2016; Nishitani et al. 2021; Seok and Sohn 2018; Tavakol et al. 2021; Wang et al. 2016; Xie, et al. 2020). Related questions should be investigated in the future.

In summary, the present study revealed that the left precentral and the left insula can be potential brain regions determining an individual’s antisaccade cost. In other words, these two brain regions are important for flexible oculomotor control. Especially, the left precentral was involved in the saccade execution functionally, while the left insula was not. Combining with the task-state fMRI results, we argue that the insula influences attention shift to saccade target before the saccade initiation via its role in attentional capture, while the precentral influences the saccade execution progress. The role of the insula may be limited to saccade task designs where the saccade target is capable of capture attention in a bottom-up manner, but the role of the precentral gyrus may be robust in flexible control in the oculomotor domain.

## Supplementary Information

Below is the link to the electronic supplementary material.

Supplementary file1 (DOCX 703 KB)

## Data Availability

Data are available from the corresponding author on reasonable request. Sharing and reuse of data require the written permission of the corresponding author.
