# Supplementary material for: Structural and functional MRI evidence for significant contribution of precentral gyrus to flexible oculomotor control: evidence from the antisaccade task
Source: Brain Struct Funct. 2022 Sep 1;227(8):2623–32. doi: 10.1007/s00429-022-02557-z (PMC9618498; doi:10.1007/s00429-022-02557-z)
Supplement: Supplementary file 1 — Supplementary file1 (DOCX 703 KB) [file 429_2022_2557_MOESM1_ESM.docx]

Table S1. Brain regions whose GMV was correlated with error rates of saccades.

| **brain region** | **cluster**  **（voxels）** | **peak MNI coordinates** | | | **T value** |
| --- | --- | --- | --- | --- | --- |
|  |  | x | y | z |  |
| ***Antisaccades Error Rate***  *Positively correlated areas* |  |  |  |  |  |
| R Cerebellum | 349 | 27 | -24 | -30 | 4.75 |
|  |  | 23 | -33 | -18 |  |
| R Middle Frontal Gyrus | 65 | 44 | 18 | 35 | 4.21 |
| L Inferior Occipital Gyrus | 85 | -44 | -72 | -5 | 4.19 |
| R Superior Temporal Gyrus | 135 | 51 | -18 | -2 | 4.01 |
| R Precuneus | 60 | 9 | -62 | 26 | 3.61 |
|  |  | 15 | -51 | 21 |  |
| R Lingual | 64 | 29 | -54 | -3 | 3.56 |
| L Calcarine | 30 | -2 | -86 | 3 | 3.48 |
| L Cerebellum | 33 | -23 | -44 | -57 | 3.41 |
|  |  |  |  |  |  |
| ***Prosaccades Error Rate***  *Positively correlated areas* |  |  |  |  |  |
| L Inferior Occipital Gyrus | 204 | -44 | -78 | -6 | 4.63 |
| R Superior Temporal Gyrus | 37 | 69 | -12 | -11 | 3.82 |
| L Calcarine | 64 | -3 | -93 | -11 | 3.79 |
|  |  | -12 | -93 | -18 |  |
| L Middle Occipital Gyrus | 92 | -44 | -75 | 14 | 3.75 |
| *Negatively correlated areas* |  |  |  |  |  |
| R Superior Occipital Gyrus | 208 | 23 | -84 | 41 | 4.15 |
|  |  | 14 | -80 | 47 |  |

Note: MNI, Montreal Neurological Institute; L: left; R: right. Significance levels were set at of p < 0.001 (uncorrected) and a minimum cluster size of 30 voxels.

Table S2. Brain regions whose GMV was positively correlated with antisaccade cost at the significance level of p < 0.001 (uncorrected) in addition to those survived the FDR correction.

| **brain region** | **cluster**  **（voxels）** | **peak MNI coordinates** | | | **T value** |
| --- | --- | --- | --- | --- | --- |
|  |  | x | y | z |  |
| L Cerebelum | 265 | -5 | -65 | -21 | 4.31 |
|  |  | 5 | -65 | -23 |  |
| R Putamen | 152 | 33 | 3 | 0 | 4.13 |
|  |  | 26 | 23 | -5 |  |
| R Fusiform | 173 | 24 | -47 | -15 | 3.94 |
|  |  | 18 | -59 | -15 |  |
| R Paracentral_Lobule | 36 | 11 | -23 | 74 | 3.59 |

Note: MNI, Montreal Neurological Institute; L: left; R: right. Significance levels were set at of p < 0.001 (uncorrected) and a minimum cluster size of 30 voxels.


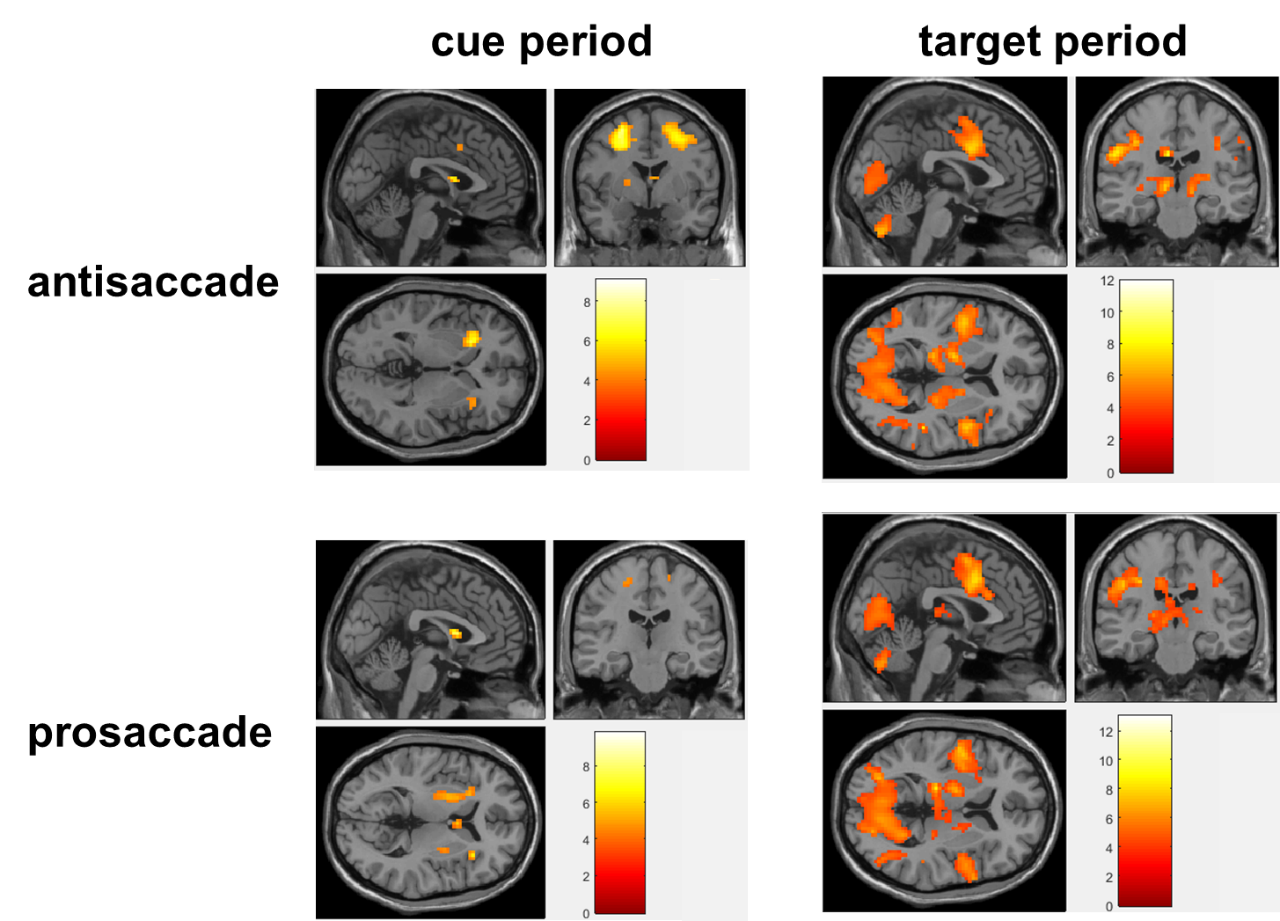


Figure S1. brain activation during the cue period and target period at the significance level of uncorrected p < 0.001, cluster size > 10 voxels.
